# Carbohydrate ingestion induces differential autonomic dysregulation in normal-tension glaucoma and primary open angle glaucoma

**DOI:** 10.1371/journal.pone.0198432

**Published:** 2018-06-07

**Authors:** Lei Cao, Stuart L. Graham, Paul M. Pilowsky

**Affiliations:** 1 The Heart Research Institute, The University of Sydney, Newtown, NSW, Australia; 2 Australian School of Advanced Medicine, Macquarie University, North Ryde, NSW, Australia; Tokai University, JAPAN

## Abstract

**Background:**

It is reported that glaucoma may be associated with vascular dysregulation. Normal tension glaucoma (NTG) and primary open angle glaucoma (POAG), which feature different intraocular pressure levels, may manifest *differential* features of systemic autonomic dysregulation.

**Methods and results:**

We investigated autonomic regulation to carbohydrate ingestion and postural change in 37 glaucoma patients (19 NTG and 18 POAG) and 36 controls. Subjects were age and gender-matched, normotensive, and had normal comparable insulin sensitivity. Continuous finger arterial pressure and ECG was recorded in supine and standing positions before and after carbohydrate ingestion. Low frequency (LF, 0.04–0.15Hz) and high frequency (HF, 0.15–0.4Hz) spectral power of heart rate and systolic blood pressure variability (HRV and SBPV) were calculated to estimate sympathovagal function. Overall comparison glaucoma (N = 37) and controls (N = 36) showed an increased sympathetic excitation, vagal withdrawal and unstable mean arterial pressure after carbohydrate ingestion in glaucoma patients. Glaucoma severity by retinal nerve fibre layer (RNFL) thickness is positively correlated to autonomic responses (HRV LF power and HF power in normalised units (nu), and HRV LF/HF ratio) after carbohydrate ingestion. Early (30 minutes) following carbohydrate ingestion, SBP LF power and HRV parameters remained unchanged in controls; while POAG showed abnormal autonomic responses, with a paradoxical vagal enhancement (increased HRV HF power in nu) and sympathetic inhibition (decreased HRV LF power nu and HRV LF/HF ratio), and associated hypotension. Later (60–120 minutes) following carbohydrate ingestion, HRV parameters remained unaltered in controls; whereas NTG manifested vagal withdrawal (reduced HRV HF power nu) and sympathetic hyper-responsiveness (increased HRV LF power nu and HRV LF/HF ratio), despite increased SBP LF power in both controls and NTG. Both NTG and POAG exhibited attenuated autonomic responses to postural stress.

**Conclusions:**

NTG and POAG both manifest some systemic autonomic cardiovascular dysregulation. However, the two forms of glaucoma respond *differentially* to carbohydrate ingestion, irrespective of insulin resistance.

## Introduction

Glaucoma is one of the most common causes of blindness, with a loss of retinal ganglion cells leading to visual field defects [1]. Growing evidence indicates that vascular risk underlies the pathogenesis of glaucoma [2], regardless of the pathological role of elevated intraocular pressure (IOP) [3, 4]. From a vascular perspective the eye (retina) provides a window to the cardiovascular system, and often manifests features of target organ damage of cardiovascular disease [5]. In clinical practice, primary open angle glaucoma (POAG) refers to glaucoma patients with elevated IOP (> 21mmHg); whereas those with IOP always < 21mmHg are termed normal tension glaucoma (NTG). While it remains controversial whether the two types are distinct entities, but rather more likely a spectrum of glaucomatous disease, it has been suggested that they may share a similar pathogenic pathway from a vascular perspective [4, 6–8].

Systemic autonomic dysfunction (different from the abnormality of autoregulation of ocular blood flow [5, 9]) is associated with metabolic syndrome [10], and may be a harbinger of cardiovascular disorders, such as hypertension and diabetes [11–14]. Metabolic syndrome contributes to the development and progression of cardiovascular disease, and this is similarly recognised in glaucoma [15]. More recent evidence shows that calorie restriction may be protective in the elderly, and reduces the incidence of glaucoma [16, 17]. Our recent study demonstrates that carbohydrate-rich meal ingestion, as a cardiovascular risk, induces abnormal sympathetic activation and vagal inhibition in older men and women [18]. A carbohydrate ingestion-mediated neuro-pathogenic role in glaucoma has not yet been studied. Furthermore, it has been previously reported that NTG and POAG patients exhibit similar autonomic dysfunction during laboratory baroreceptor stimuli [19], and under endogenous circadian rhythm [6]. From a vascular perspective, different mechanisms underlying the pathogenesis of NTG and POAG have been reported. For example, autonomic failure is associated with instability of systemic perfusion pressure and ocular flow in POAG [9]; whereas sympathovagal imbalance [20] is associated with excessive central visual field progression [21] in NTG. While clinicians now recognise vascular risk factors in glaucoma, no study has investigated the distinguishing features of systemic autonomic dysfunction in NTG and POAG. The carbohydrate ingestion induced autonomic responses provide opportunities to examine, firstly, baroreceptor-mediated reflex response to hemodynamic perturbation [18] induced by splanchnic hyperaemia early after eating [22]; and secondly, in the later phase of postprandial state, carbohydrate ingestion-mediated hyperinsulinemia becomes evident [23, 24]; and the central action of insulin [25, 26], together with other neurotransmitters [27, 28], evokes changes in sympathetic and vagal outflow [18, 29]. We therefore hypothesized that *differential* features of autonomic dysfunction may exist in the two forms of glaucoma, and correlate with the mechanism of the entire glaucomatous optic neuropathy process in NTG and POAG. The current study aimed to evaluate (1) whether or not daily stressors such as carbohydrate ingestion and postural challenge (5 minutes quiet standing used in this study) may induce autonomic dysregulation in glaucoma patients, and (2) whether or not there are distinct features of autonomic dysregulation following these daily stressors in NTG and POAG.

Power spectral analysis of blood pressure variability [30], and heart rate variability [31] was used to estimate autonomic modulation. HRV LF power (nu) and HRV HF power (nu), and HRV LF/HF ratio as indexes of cardiac sympathovagal responses were used [30–33]. SBP LF power served as a surrogate marker of sympathetic modulation to the entire peripheral vasculature [30, 33]; Spontaneous heart rate baroreflex responses to BP change were calculated to estimate baroreflex sensitivity [34].

## Materials and methods

### Study participants

The study was approved by the Ethics Committee at Macquarie University, NSW, Australia (Ethics Approval Ref: 5201100552). All subjects provided written informed consent.

Previously diagnosed glaucoma patients were recruited by a consultant ophthalmologist (SLG) and included both POAG and NTG patients. In addition, glaucoma patients and control volunteers were recruited from the general population via an advertisement in the local newspapers and the newsletter of Glaucoma Australia. Accuracy of diagnosis was confirmed from medical records and a retinal nerve fibre layer (RNFL) scan (Spectralis OCT, Heidelberg Eng, Germany) after the experimental study. All patients had been diagnosed with either form of open angle glaucoma for at least 12 months. Control volunteers were defined as normal based on medical history, ophthalmic examination, and a RNFL scan. For any control subjects who were suspect in RNFL scan results, further clinical investigations were conducted by consultant ophthalmologist (SLG) to confirm, or exclude, the diagnosis of glaucoma. All participants fulfilled inclusion criteria: age of 50–80 years; no hypertension, diabetes or metabolic syndrome; no history of heart disease. Subjects were not prescribed oral vasoactive medications (angiotensin-converting enzyme inhibitors, angiotensin receptor blockers, α-or β- adrenergic blocker, calcium channel blocker, and diuretics). All female subjects were postmenopausal (defined as at least one entire year since last menstruation, and not taking hormone replacement therapy). Exclusion criteria were: cardiac arrhythmia identified during the experiment, i.e. atrial fibrillation and/or frequent ectopic beats. Two volunteers could not be differentiated as either early glaucoma or normal based on clinical investigations and were excluded. In total, 36 control subjects and 37 glaucoma subjects (NTG, N = 19; POAG, N = 18) were eligible and entered the study.

Subjects were instructed not to drink water 1.5 hours prior to experiments, and to abstain from caffeinated beverages and food for 12 h, alcohol for 24 h, and moderate or strenuous physical activity for 48 h prior to the experiment.

### Clinical investigations

Anthropometric measurements comprised body weight, BMI, waist circumference, and waist/hip ratio. Fasting blood sample was collected from each subject for measures of renal function, lipid profile, plasma glucose and insulin levels. HOMA (the homeostasis model assessment) (HOMA = (fasting glucose mg/dL X fasting insulin μU/mL) / 405) [35] and QUICKI (quantitative insulin sensitivity check index) (QUICKI = 1 / (log(fasting insulin μU/mL) + log(fasting glucose mg/dL)) [36] are calculated as accurate surrogate markers used for evaluating insulin sensitivity.

### Recordings

As previously described [18, 29], electrocardiogram (ECG) and BP (finger photoplethysmography; Finometer Pro, Ohmeda, Amsterdam, Holland) were measured continuously. ECG was sampled at 1000 Hz and stored for off-line analysis (LabChart 7.2 and Powerlab8/30, ADInstruments, Bella Vista, Sydney, NSW, Australia). BP waveform files were recorded at a sampling rate of 200 Hz for further power spectral analysis. The subject's left arm was placed with the left hand (testing hand) at the level of the heart at all times (the testing hand was maintained at the heart level while standing up by a 90-degree elbow flexion). Brachial arterial blood pressure was recorded from the right arm with an automated sphygmomanometer (Microlife A100 PLUS, AG, 9443 Widnau, Switzerland) to confirm the accuracy of the Finometer measurements of absolute blood pressure. All other hemodynamic variables were downloaded with a Finolink and derived from Beatscope software (FMS, Finapres Medical Systems BV, Amsterdam, The Netherlands). Details of the methodology used by the Finometer software (“beatscope”) to calculate different haemodynamic parameters are available from http://www.finapres.com/site/index.php [37].

### Experimental protocol

The detailed experimental protocol is as described in our previous studies [18, 29]. Briefly, subjects were fasted overnight and experiments were conducted from 0900 to 1230. The laboratory temperature is centrally air-conditioned and maintained at 23°C throughout experiments. All experiments were undertaken in a quiet semi-dark room. Baseline measurements of all parameters (brachial BP, and 5 minutes of ECG and Finger blood pressure recording) were obtained in the supine state after 20 minutes duration of supine rest. Subjects then stood, and after 2 minutes to allow for hemodynamic equilibration, data was recorded again for 5 minutes standing position.

**Fig 1:**
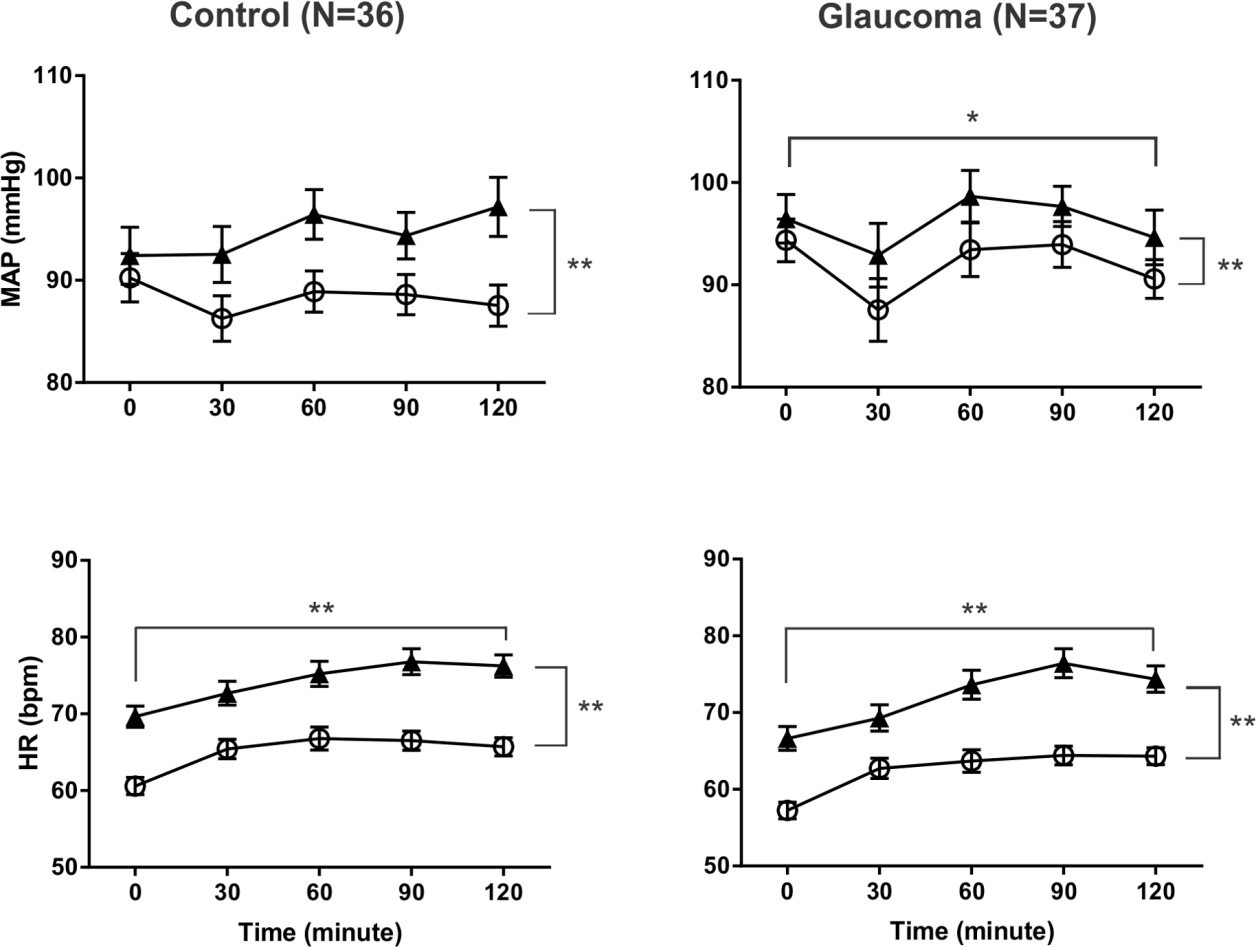
Hemodynamic changes in response to carbohydrate ingestion and postural stress (glaucoma N = 37 vs controls N = 36). From fasting (0 min) to postprandial state (30, 60, 90, and 120min), MAP maintained stable in controls, but was unstable in glaucoma patients; HR increased in both control and glaucoma subjects. (*P<0.05, **P<0.01, effect of carbohydrate ingestion; two-way ANOVA). From supine (○) to standing (▲), both MAP and HR increased in glaucoma and control groups. (**P<0.01, effect of postural change before and after carbohydrate ingestion; two-way ANOVA).

Subjects were then fed a standard breakfast, comprising 30g ‘Weet-Bix Bites’ (wild berry) (Sanitarium Health and Wellbeing, Australia), 100ml ‘Original Milk’ (Dairy Farmer Pty Ltd, Australia), 170ml Low Fat Fruit Yogurt (Dairy Farmer Pty Ltd; Australia), 200ml Orange Juice (The Daily Juice Company, Australia), one medium-sized banana (all sourced from Woolworth Ltd, Australia). The food formula is a 600kcal carbohydrate-rich mixed meal (semi-liquid): 118g carbohydrate (including 85g sugar) (78%), 20g protein (13%), 6g fat (8%), sodium 300mg. The carbohydrate-rich meal was consumed within 10–12 minutes in a normal sitting position. The first 30-minute time interval was defined as starting from the first mouth of food intake (10–12 minutes of eating time), with subsequent 18–20 minutes post-prandial supine resting state. Postprandial recordings were then conducted and repeated at each beginning of 30-minute time-interval for a further 2 hours. Each time-interval incorporates 12 minutes recording time (5 minutes lying and 7 minutes standing), followed by 18 minutes in the supine resting state (see a diagram (Fig 1.) in our previous study [29]).

### Data analysis

Power spectral analysis of RR interval was calculated with the HRV module in the commercial software of LabChart. (LabChart 7.2, ADInstruments, Bella Vista, Sydney, NSW, Australia). Power spectrum analysis of systolic blood pressure (SBP) was performed with Spike2 software (Cambridge Electronic Design Limited, Cambridge, England). All analyses were performed from stable haemodynamic regions with a duration of 5 minutes that were free of ectopic beats and any technical artefacts.

A fast Fourier Transform (Hanning window; 512 block size) was applied to the RR interval and SBP in time series to obtain power spectral estimates of HRV and BPV [30, 31]. The low-frequency refers to 0.04–0.15 Hz, and high-frequency refers to 0.15–0.40 Hz. Signal powers of each frequency band were calculated as integrals under the respective power spectral density functions and expressed in absolute units (ms^2^) and normalised units for HRV, and absolute units for BPV (mmHg^2^).

Spontaneous baroreceptor reflex sensitivity (RR interval to systolic BP) was evaluated with HemoLab software: http://haraldstauss.com/HemoLab/HemoLab.php, using the sequence method that identifies sequences of four or more heart beats, where BP and pulse interval change in the same direction [38]; for example, rises in BP corresponding increases in pulse intervals, with minimal SBP 1mmHg and pulse interval 6ms as threshold changes in humans [39]. Only sequences with a coefficient of determination (*R*^2^) > 0.8 were included in the BRS analysis. A delay of 0–2 physiological beat cycle(s) between systolic blood pressure and pulse interval was used to provide the most representative estimates of BRS. In a very small number of cases, in particular, while hemodynamic perturbation peaks [22], a delay of 3–5 beats was used to achieve a larger number of slopes for optimal estimates of BRS [18, 40, 41].

### Statistical analysis

All statistical analyses were calculated using GraphPad Prism software (version 7). Subject profiles, clinical characteristics and baseline hemodynamic and autonomic data were evaluated by using unpaired *t* test or Fisher’s exact tests between study groups. Variable’s comparison (from lying to standing) within groups were evaluated by using paired *t* test. Statistical significance for hemodynamic and autonomic responses to postural stress and meal ingestion was evaluated using two-way ANOVA (repeated measures), followed by Bonferroni post-hoc corrections. To clarify the meal ingestion effect in either supine or standing position between study groups, unpaired *t*-tests were used to compare HRV parameters’ changes from fasting (pre-ingestion) to early after eating (30min), or from fasting to later postprandial state (maximal response at 90min). Pearson correlation coefficient (Pearson’ *r*) was used to measure linear correlation between glaucoma severity and autonomic parameters. Power calculations were undertaken in order to ensure that biologically important differences between the means could be detected at the 80% power [18, 29]. In this study, same experimental protocol was applied to same age cohort with a similar sample size, in comparison to our previous study [18]. A *P* value <0.05 was regarded as statistically significant. Data are presented as mean ± SEM in figures and tables.

## Results

### Clinical characteristics of study groups

There were no statistically significant age and gender differences between 36 control subjects (14 males and 22 females) and 37 glaucoma patients (14 males and 23 females) (Fisher’s exact test, *P* = 1), as well as between each two group comparison of controls (N = 36), NTG (N = 19), and POAG (N = 18) (Table 1). BMI, waist/hip ratio, lipid profile, renal function, and fasting blood glucose and insulin levels were comparable between each of two groups of controls, NTG and POAG (Table 1). HOMA and QUICKI as surrogate markers of insulin sensitivity showed no difference between each group comparison (Table 1).

**Table 1:** Subjects’ general profiles.

	Control (N = 36)	NTG (N = 19)	POAG (N = 18)	*P* value		
				Control vs NTG	Control vs POAG	NTG vs POAG
Age (years)	63.83 ± 1.38	62.58 ± 2.07	66.33 ± 1.66	0.61	0.28	0.17
Sex (female, N, %)	22, 61%	13, 68%	10, 56%	0.77	0.77	0.51
BMI (kg/m^2^)	24.95 ± 0.81	24.01 ± 0.76	26.04 ± 0.97	0.46	0.42	0.11
Waist/hip ratio	0.90 ± 0.01	0.86 ± 0.02	0.89 ± 0.02	0.13	0.61	0.48
Serum Triglycerides (mmol/L)	1.08 ± 0.09	1.25 ± 0.21	1.37 ± 0.17	0.36	0.10	0.67
Serum Total Cholesterol (mmol/L)	5.45 ± 0.18	5.54 ± 0.16	5.90 ± 0.30	0.71	0.14	0.29
Serum Creatinine level (mmol/L)	70.56 ± 2.14	68.21 ± 2.32	69.72 ± 2.44	0.49	0.81	0.66
Corrected Glomerular Filtration Rate (cGFR)(ml/min/1.73m^2^)	85.76 ± 3.99	84.09 ± 4.51	89.39 ± 4.61	0.79	0.58	0.42
Fasting blood sugar level (mmol/L)	5.29 ± 0.08	5.26 ± 0.09	5.65 ± 0.23	0.83	0.08	0.11
Fasting serum insulin level (mU/L)	6.75 ± 0.57	7.32 ± 0.90	6.89 ± 0.86	0.58	0.89	0.73
HOMA = (fasting glucose mg/dL X fasting insulin μU/mL) / 405	1.62 ± 0.15	1.73 ± 0.22	1.80 ± 0.28	0.70	0.55	0.84
QUICKI = 1 / (log(fasting insulin μU/mL) + log(fasting glucose mg/dL)	0.364 ± 0.0045	0.361 ± 0.0069	0.361 ± 0.0075	0.73	0.76	0.99

Data shown as Mean ± SEM. *P* value indicates group comparison using unpaired *t*-test; *P*<0.05 is defined as statistical significance.

The glaucoma severity included a range from mild to advanced disease, as shown by the data in Table 2. There was no significant difference in severity for comparison between POAG and NTG for all visual field and RNFL parameters (Table 2).

**Table 2:** Subjects’ glaucoma severity.

		Visual Field Mean Deviation Right Eye	Visual Field Mean Deviation Pattern Standard Deviation Right Eye	Visual Field Index Right Eye	Visual Field Mean Deviation Left Eye	Visual Field Mean Deviation Pattern Standard Deviation Left Eye	Visual Field Index Left Eye	Retinal nerve fibre layer thickness Right Eye	Retinal nerve fibre layer thickness Left Eye
		DB		%	DB		%	μm	μm
All Glaucoma	Mean	-4.42	6.43	86.67	-4.64	5.54	87.0	70.8	72.8
N = 37	SD +/-	4.50	4.89	14.15	5.98	4.84	17.5	12.7	14.2
	Max	1.29	15.59	100	1.33	15.58	100	100	101
	Min	-16.30	1.36	55	-24.63	1.16	26	52	45
POAG	Mean	-3.85	5.06	88.00	-4.33	5.61	86.9	69.8	71.7
N = 18	SD +/-	5.66	4.68	16.44	5.53	4.92	15.4	13.7	13.8
	Max	1.29	15.02	100	0.21	13.38	99	100	101
	Min	-16.30	1.36	55	-18.66	1.52	51	52	45
NTG	Mean	-4.81	7.38	85.75	-4.86	5.49	87.1	71.8	73.9
N = 19	SD +/-	3.65	4.95	12.84	6.44	4.94	19.3	12.1	15.1
	Max	0.98	15.59	100	1.33	15.58	100	94	97
	Min	-10.89	1.40	66	-24.63	1.16	26	58	49
*P* =		0.63	0.23	0.71	0.82	0.95	0.98	0.65	0.67

Mean, standard deviation and range for Visual field indices and OCT RNFL thickness for right and left eyes of all subjects *P* = Students *t* test (unpaired)

VFMD = Visual Field Mean Deviation

VF PSD = Visual Field Mean Deviation Pattern Standard Deviation

VFI = Visual Field Index

RNFL = Retinal nerve fibre layer thickness

RE = Right eye

LE = Left eye

### Baseline (pre-ingestion) hemodynamic and autonomic parameters

Pre-ingestion hemodynamic and autonomic parameters in the supine or standing position did not differ between study groups (Table 3).

**Table 3:** Baseline (pre-ingestion) hemodynamic and autonomic data.

	Control (N = 36)	NTG (N = 19)	POAG (N = 18)	*P* value			
				Control vs NTG	Control vs POAG	NTG vs POAG	Supine vs Standing
**Supine position**							
Resting SBP (mmHg)	122.5 ± 2.52	124.7 ± 2.56	126.5 ± 3.78	0.57	0.37	0.70	
Resting DBP (mmHg)	73.50 ± 1.50	76.89 ± 2.00	77.44 ± 2.80	0.18	0.18	0.87	
Resting MAP (mmHg)	89.83 ± 1.73	92.84 ± 1.92	93.80 ± 3.04	0.28	0.23	0.79	
Resting HR (bpm)	60.72 ± 1.21	57.68 ± 1.68	58.78 ± 1.86	0.15	0.37	0.66	
SBP LF power (mmHg^2^)	7.04 ± 1.06	11.13 ± 2.44	9.30 ± 2.04	0.08	0.28	0.57	
HRV LF power (ms^2^)	647.0 ± 156.9	499.6 ± 167.7	1077.0 ± 415.3	0.56	0.25	0.20	
HRV LF nu	52.80 ± 3.71	47.77 ± 4.94	59.26 ± 5.31	0.42	0.32	0.12	
HRV HF power (ms^2^)	603.9 ± 204.4	546.3 ± 127.7	775.8 ± 397.3	0.85	0.67	0.58	
HRV HF nu	41.27 ± 3.02	45.24 ± 3.82	35.49 ± 4.29	0.43	0.27	0.10	
HRV LF/HF ratio	1.82 ± 0.23	1.44 ± 0.28	2.94 ± 0.78	0.33	0.09	0.07	
BRS (ms/mmHg)	14.61 ± 1.68	16.45 ± 3.28	11.88 ± 1.55	0.58	0.29	0.20	
**Standing position**							
Resting SBP (mmHg)	130.6 ± 3.04	130.9 ± 2.64	139.1 ± 3.39	0.95	0.09	0.06	# §
Resting DBP (mmHg)	83.47 ± 1.52	84.79 ± 1.87	87.83 ± 2.39	0.60	0.12	0.32	## ϮϮ §§
Resting MAP (mmHg)	99.18 ± 1.88	100.2 ± 2.00	104.9 ± 2.49	0.74	0.08	0.14	## Ϯ §§
Resting HR (bpm)	70.14 ± 1.46	66.16 ± 2.30	68.56 ± 2.34	0.13	0.55	0.47	## ϮϮ §§
SBP LF power (mmHg^2^)	21.18 ± 2.76	19.57 ± 2.74	16.65 ± 2.17	0.72	0.28	0.41	## Ϯ §
HRV LF power (ms^2^)	512.0 ± 108.7	448.3 ± 77.91	258.3 ± 62.17	0.69	0.12	0.07	
HRV LF nu	61.52 ± 4.35	51.40 ± 6.29	62.85 ± 4.84	0.18	0.85	0.16	##
HRV HF power (ms^2^)	668.4 ± 326.4	537.0 ± 193.7	131.5 ± 37.10	0.78	0.25	0.05	
HRV HF nu	29.50 ± 3.02	36.63 ± 4.49	26.43 ± 2.95	0.18	0.52	0.07	##
HRV LF/HF ratio	4.021 ± 0.65	3.520 ± 1.39	3.301 ± 0.53	0.71	0.48	0.89	##
BRS (ms/mmHg)	8.79 ± 1.70	7.29 ± 0.95	7.68 ± 1.94	0.56	0.69	0.86	# Ϯ

Data shown as Mean ± SEM. *P* < 0.05 is defined as statistical significance.

*P* value indicates group comparison using unpaired *t*-test (Control vs NTG, Control vs POAG, and NTG vs POAG); variable’s change from supine to standing position within each group using paired *t*-test (# P<0.05, ## P<0.01 for Control; Ϯ P<0.05, ϮϮ P<0.01 for NTG; § P<0.05, §§ P<0.01 for POAG).

In the fasting state (pre-ingestion), from supine to standing position, SBP, DBP, MAP and HR increased in all three groups, except for an altered SBP in NTG group (Table 3). In response to postural stress, SBP LF power increased in all three groups (*P*<0.01 in controls, *P*<0.05 in NTG and POAG). HRV HF power nu reduced, and HRV LF power nu and HRV LF/HF ratio increased in control subjects only (all *P*<0.01); HRV parameters did not change in NTG and POAG patients. BRS decreased in controls and NTG (both *P*<0.05), but not in POAG (Table 3).

### Hemodynamic and autonomic responses to postural stress and carbohydrate ingestion in controls (N = 36) and open angle glaucoma patients (N = 37)

**Fig 2:**
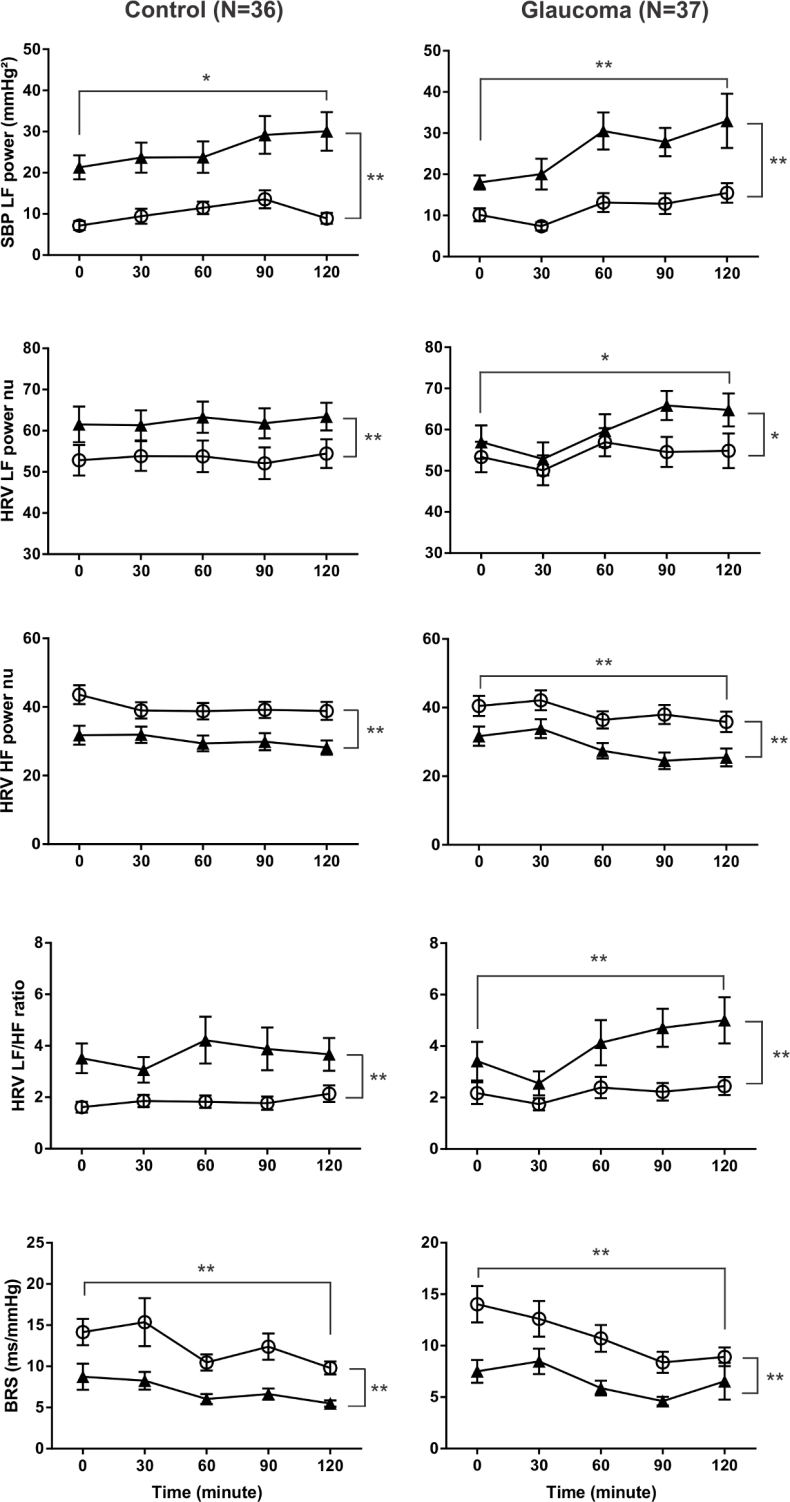
Autonomic changes in response to carbohydrate ingestion and postural stress (glaucoma N = 37 vs controls N = 36). From fasting (0 min) to postprandial state (30, 60, 90, 120 min), HRV LF nu increased, HRV HF nu decreased, and HRV LF/HF increased in glaucoma patients; whereas HRV parameters remained unaltered in controls, albeit an increase in HRV LF nu. SBP LF power increased and BRS reduced in both glaucoma and control subjects. (*P<0.05, **P<0.01, effect of carbohydrate ingestion; two-way ANOVA). From supine (○) to standing (▲) position, SBP LF power, BRS and all HRV parameters showed comparable changes in glaucoma and control groups. (*P< 0.05, **P<0.01, effect of postural change before and after carbohydrate ingestion; two-way ANOVA).

In response to postural stress, MAP increased and HR increased in both control subjects and glaucoma patients (Fig 1). HRV LF nu increased, HRV HF nu decreased, and HRV LF/HF ratio increased; SBP LF power increased and BRS decreased in both control subjects and glaucoma patients (Fig 2).

In response to carbohydrate ingestion, MAP remained unaltered in controls, but fluctuated in glaucoma patients, i.e. MAP fell at 30min after meal ingestion and rose to peak level at 60min after meal ingestion (Fig 1). HR increased in both control and glaucoma subjects (Fig 1). HRV LF nu, HRV HF nu and HRV LF/HF ratio remained unchanged in controls subjects. In contrast, in glaucoma patients, HRV LF nu increased, HRV HF nu reduced, and HRV LF/HF ratio increased (Fig 2). In both control, and glaucoma subjects, SBP LF power increased, and sBRS decreased (Fig 2).

### Correlation between open angle glaucoma severity by RNFL thickness and autonomic parameters (number of XY pairs = 35)

In the pre-ingestion state (baseline), there was no correlation between RNFL thickness and HRV LF nu, HRV HF nu, HRV LF/HF ratio and SBP LF power in either supine or standing position (Table 4). After carbohydrate ingestion, in standing position, but not supine position, there was a weak but significant linear correlation between RNFL thickness and HRV LF nu (R^2^ = 0.164, P<0.05), HRV HF nu (R^2^ = 0.194, P<0.01), and HRV LF/HF ration (R^2^ = 0.142, P<0.05) (Table 4).

**Table 4:** Correlation between glaucoma severity and autonomic response.

**Pre-ingestion (baseline)**		HRV LF nu	HRV HF nu	HRV LF/HF	SBP LF power
supine	R squared	0.021	0.062	0.012	0.01
	P value	0.4	0.148	0.539	0.579
standing		HRV LF nu	HRV HF nu	HRV LF/HF	SBP LF power
	R squared	0.057	0.112	0.049	0.084
	P value	0.166	0.05	0.2	0.107
**post carbohydrate ingestion**		HRV LF nu	HRV HF nu	HRV LF/HF	SBP LF power
supine	R squared	0.029	0.073	0.07	0.007
	P value	0.332	0.117	0.125	0.653
standing		HRV LF nu	HRV HF nu	HRV LF/HF	SBP LF power
	R squared	0.164	0.194	0.142	0.062
	P value	0.016*	0.008**	0.026*	0.168

R squared indicates the linear correlation between glaucoma severity by RNFL thickness and autonomic parameters (mean value of 60+90+120min postprandially)

*P< 0.05

**P<0.01

Number of XY pairs = 35

### Hemodynamic and autonomic responses to postural stress and carbohydrate ingestion in the early postprandial state (30min)

**Fig 3:**
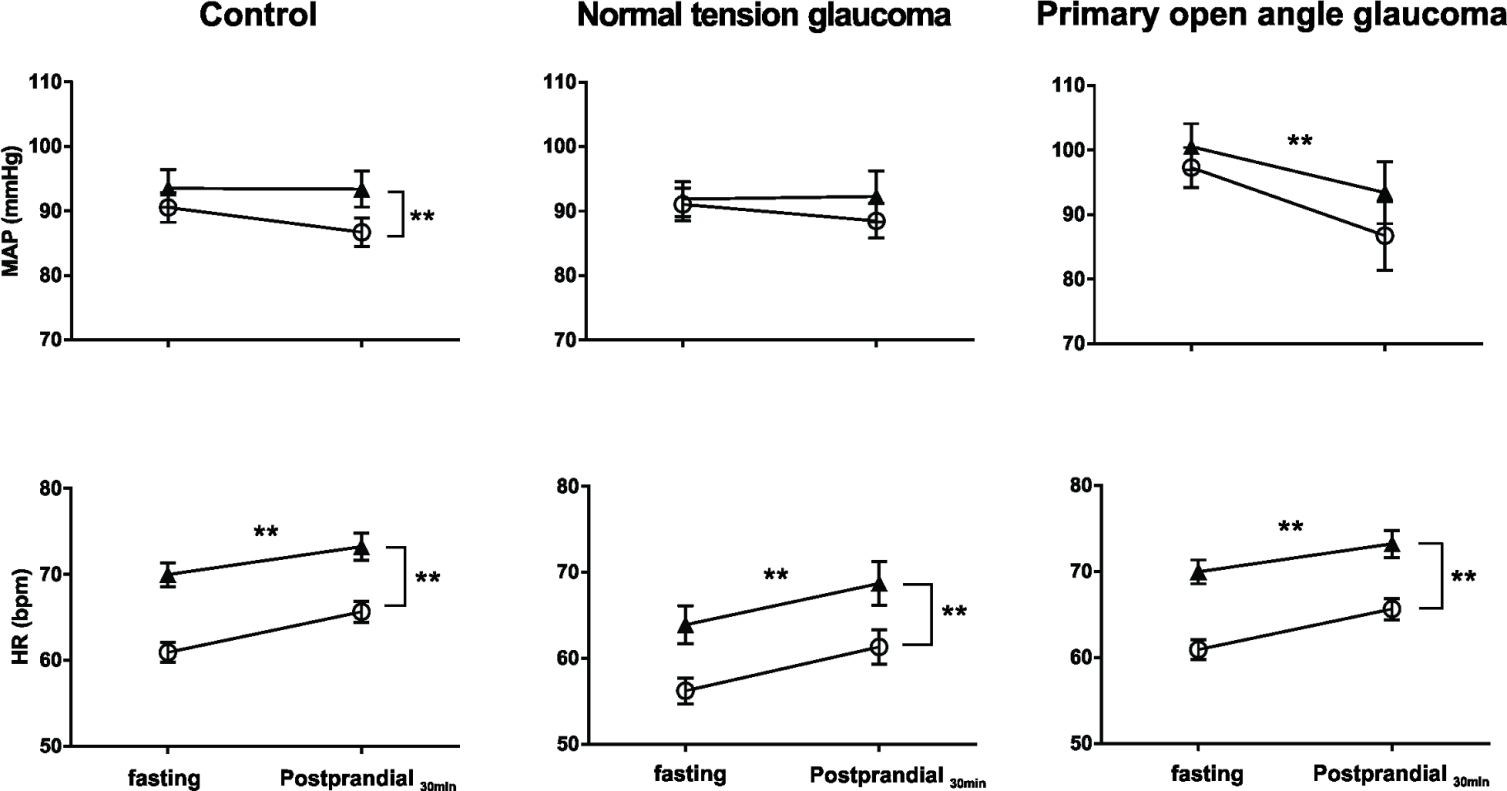
Hemodynamic responses 30 minutes following carbohydrate ingestion. MAP fell 30min after meal ingestion in POAG, whereas MAP maintained stable in control and NTG subjects. HR increased 30min after eating in all three groups. HR also increased in response to postural stress, i.e., from supine (○) to standing (▲).** P<0.01; data shown as Mean ± SEM.

**Fig 4:**
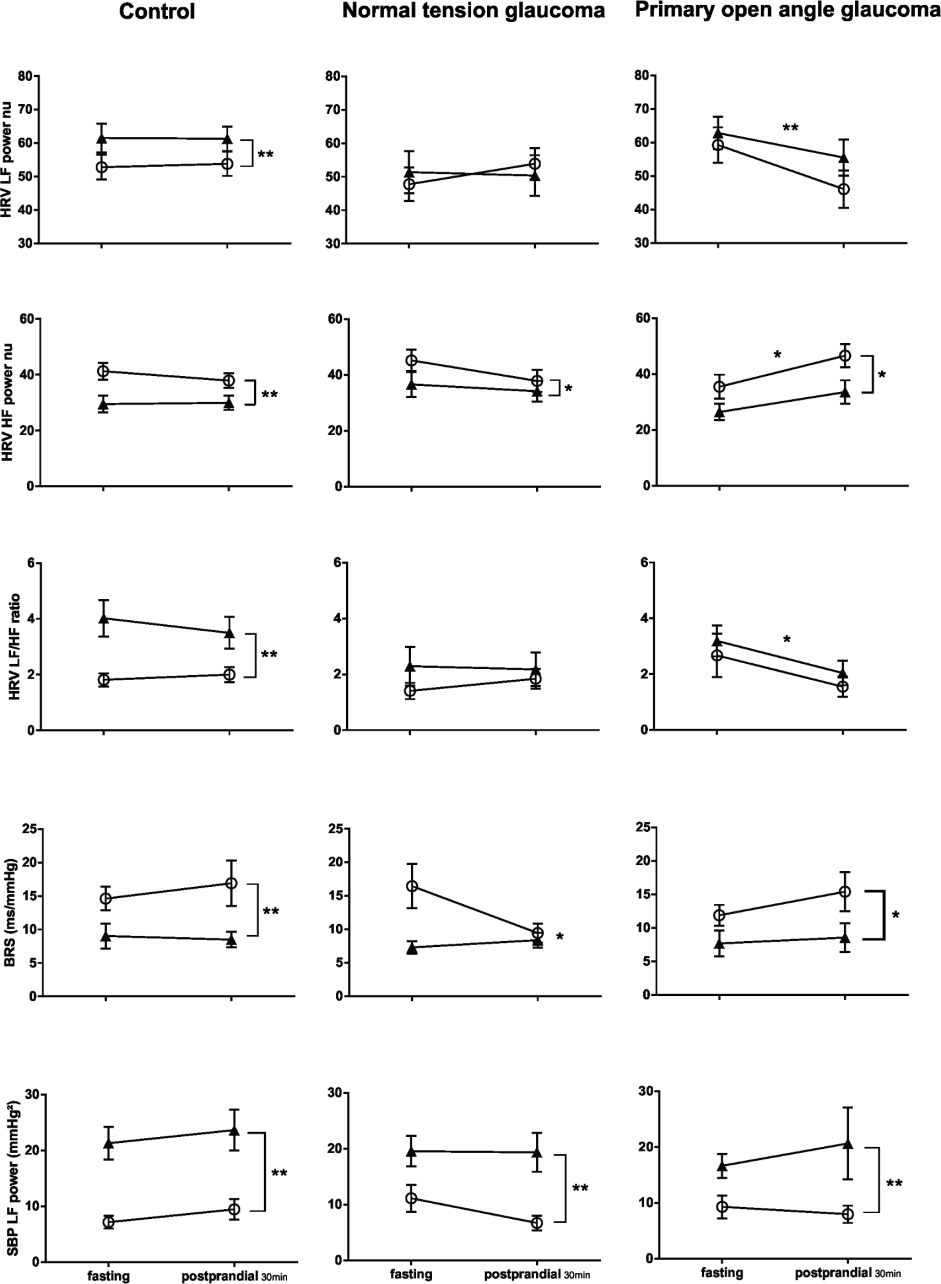
Autonomic responses 30 minutes following carbohydrate ingestion. Soon after meal ingestion, POAG exhibited a reduced HRV LF power nu, an enhanced HRV HF power nu, with a decrease in HRV LF/HF, indicating a paradoxical sympathetic and vagal outflow, i.e., autonomic failure. This autonomic response was not seen in NTG, and control subjects. In response to postural stress (from supine (○) to standing (▲)), in pre-ingestion and early fed state, HRV LF power nu increased, HRV HF power nu decreased, and HRV LF/HF increased in control subjects; whereas in NTG and POAG, autonomic response was attenuated, despite a reduction in HRV HF power nu was observed. SBP LF power and BRS changes in response to either postural stress or carbohydrate ingestion were similar among control, NTG and POAG. *P<0.05, ** P<0.01; data shown as Mean ± SEM.

In response to postural stress, MAP increased in controls (*P*<0.01), but not in NTG (*P* = 0.23) or POAG (*P* = 0.09) patients. HR increased in all three groups (all *P*<0.01) (Fig 3). HRV LF nu increased, HRV HF nu decreased and the HRV LF/HF ratio increased in controls (all *P*<0.01). Although HRV HF nu decreased in NTG and POAG groups (*P*<0.05), HRV LF nu and HRV LF/HF ratio did not change in NTG (*P* = 0.99, *P* = 0.24) and POAG (*P* = 0.26, *P* = 0.45) subjects. BRS decreased and SBP LF power increased in all groups (Fig 4).

Early after carbohydrate ingestion (30 min), in both supine and standing positions, MAP fell significantly (approx.10mmHg) in POAG patients (*P*<0.01), from 97.3mmHg to 86.7mmHg in the supine state, and from 100.5 mmHg to 93.4 mmHg during quiet standing (Fig 3). MAP remained stable in control (*P* = 0.18) and NTG (*P* = 0.77) subjects (Fig 3). HR increased in all three groups (all *P*<0.01) (Fig 3). In POAG patients, HRV HF nu increased (*P* = 0.01), and HRV LF nu and HRV LF/HF ratio decreased (*P*<0.01, *P* = 0.04) (Fig 4). In contrast, HRV LF nu, HRV HF nu and HRV LF/HF ratio remained unchanged in control subjects (*P* = 0.90, 0.59, 0.71) and NTG patients (*P* = 0.64, 0.15, 0.75) (Fig 4). SBP LF power and BRS remained unchanged in all three groups (Fig 4).

### Autonomic and hemodynamic responses to postural stress and carbohydrate ingestion in the later phase of the postprandial state (mean of 60, 90, 120min)

**Fig 5:**
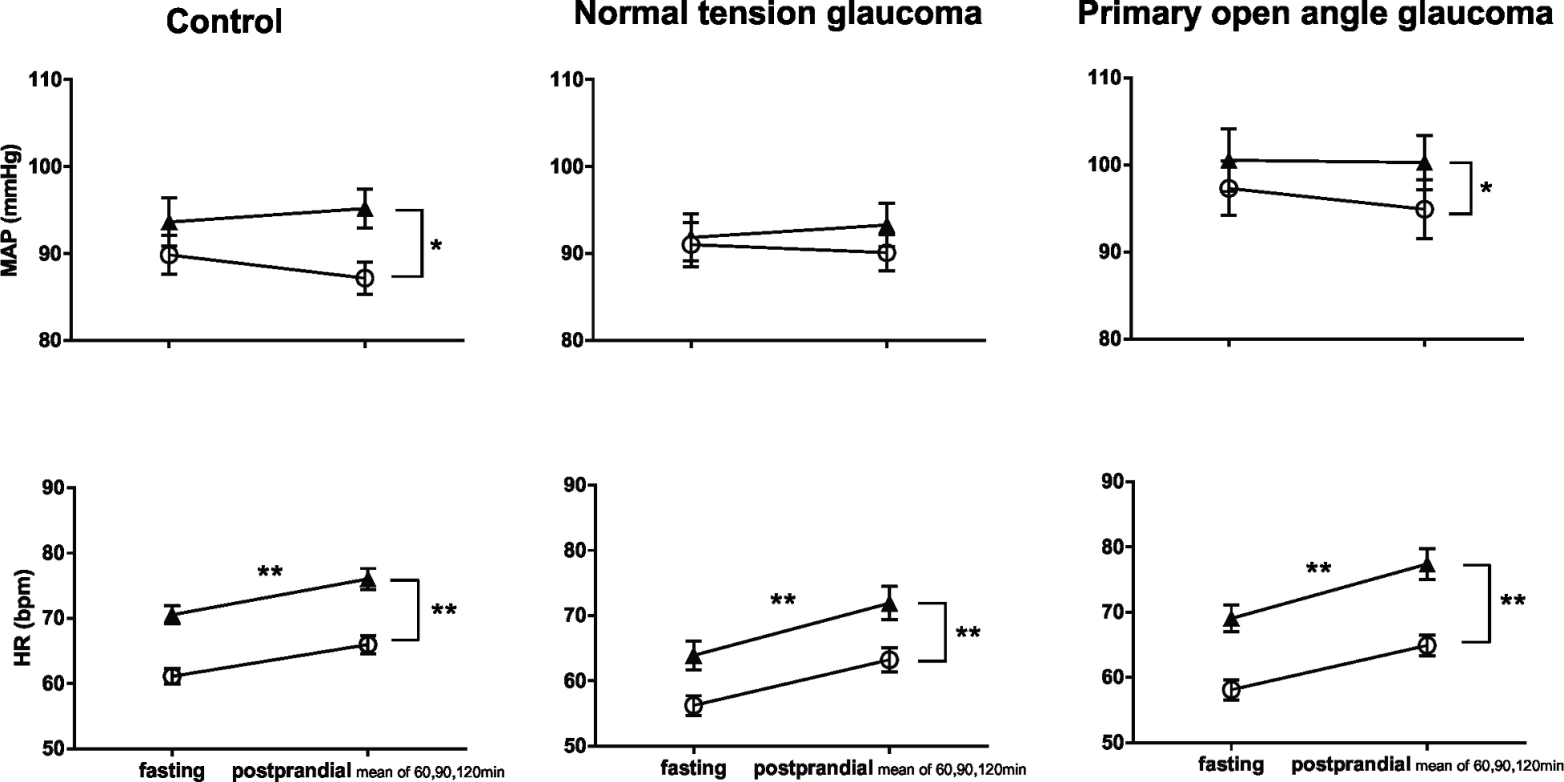
Hemodynamic responses at 60, 90 and 120 minutes following carbohydrate ingestion. Following carbohydrate ingestion, in the later phase of postprandial state, MAP maintained stable and HR increased in all three groups. In response to postural stress (from supine (○) to standing (▲)), MAP increased in control and PAOG, but not in NTG. HR increased in all three groups. *P<0.05, ** P<0.01; data shown as Mean ± SEM.

**Fig 6:**
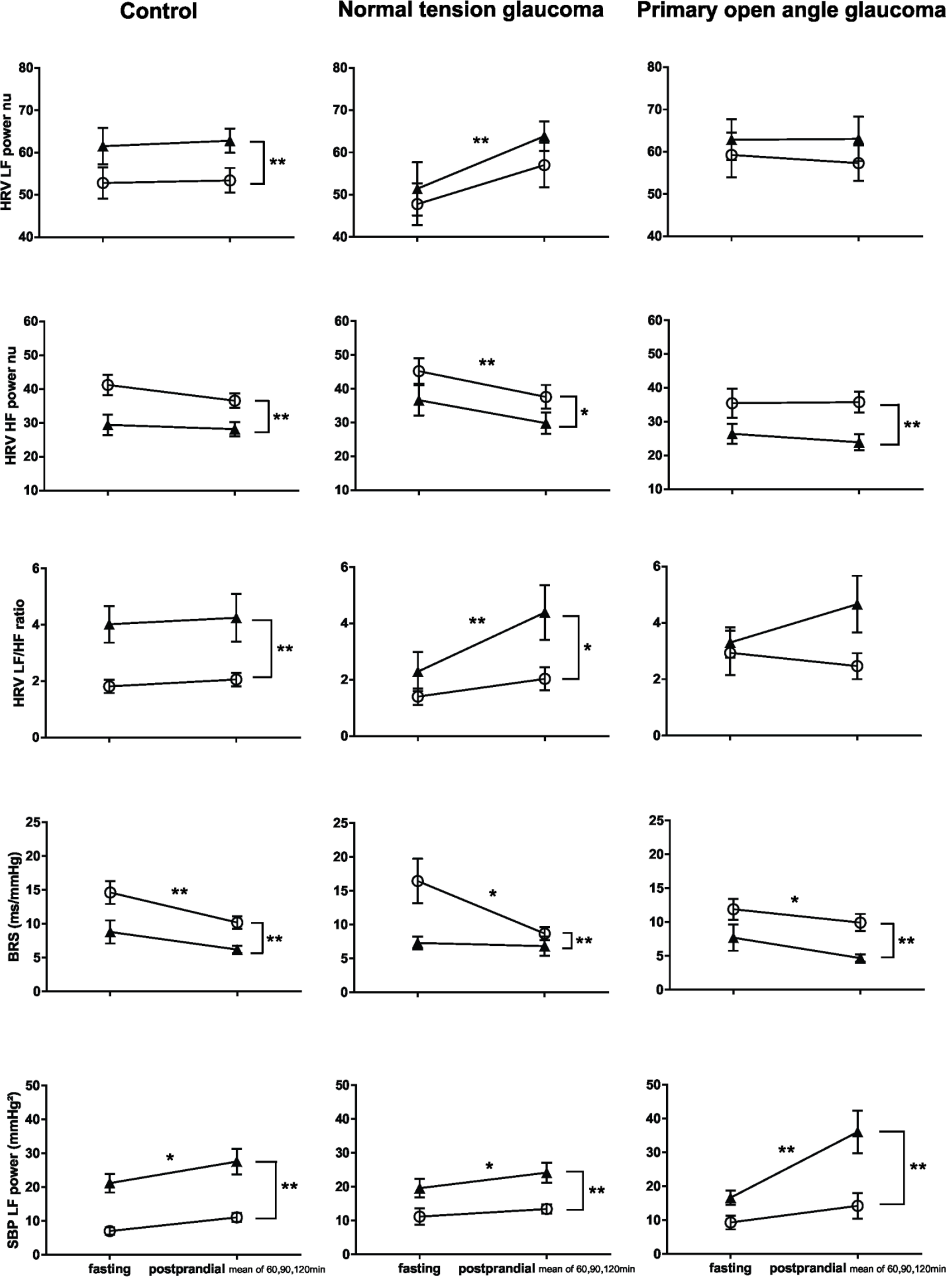
Autonomic responses at 60, 90 and 120 minutes following carbohydrate ingestion. Following carbohydrate ingestion, in the later phase of postprandial state, NTG showed an increased HRV LF power nu, a decreased HRV HF power nu, and an increased HRV LF/HF; this autonomic response is not seen in control and POAG groups. In response to postural stress (from supine (○) to standing (▲)), HRV HF power nu reduced in all three groups; whereas only control subjects exhibited an intact HRV parameters’ changes. SBP LF power and BRS changes in response to either postural stress or carbohydrate ingestion were similar among control, NTG and POAG. *P<0.05, ** P<0.01; data shown as Mean ± SEM.

In response to postural stress, MAP increased in control and POAG subjects (both *P*<0.05), but remained unchanged in the NTG group (*P* = 0.26) (Fig 5). HR increased in all three groups (all *P*<0.01) (Fig 5). HRV LF nu increased, HRV HF nu decreased and HRV LF/HF ratio increased in controls (all *P*<0.01) (Fig 6). In NTG patients, from lying to standing, HRV HF nu decreased (*P*<0.05), HRV LF nu remained unaltered (*P* = 0.19), and HRV LF/HF ratio increased (*P*<0.05). In POAG patients, HRV HF nu reduced (*P*<0.01), HRV LF nu and HRV LF/HF ratio remained unchanged (*P* = 0.84, *P* = 0.47) (Fig 6). SBP LF power increased, and BRS was reduced, in control, NTG and POAG subjects (all *P*<0.01) (Fig 6).

In response to carbohydrate ingestion in the later phase of the postprandial state, in both supine and standing positions, MAP remained stable, and HR increased in all three groups (Fig 5). HRV LF nu increased, HRV HF nu reduced, and HRV LF/HF ratio increased in NTG patients (all *P*<0.01) (Fig 6). In contrast, HRV LF nu, HRV HF nu and HRV LF/HF ratio remained unchanged in control subjects (*P* = 0.71, 0.14, 0.51) and POAG patients (*P* = 0.84, 0.70, 0.47) (Fig 6). SBP LF power increased and BRS decreased in control, NTG and POAG subjects (Fig 6).

### Group comparison of HRV parameters’ changes to meal ingestion

**Fig 7:**
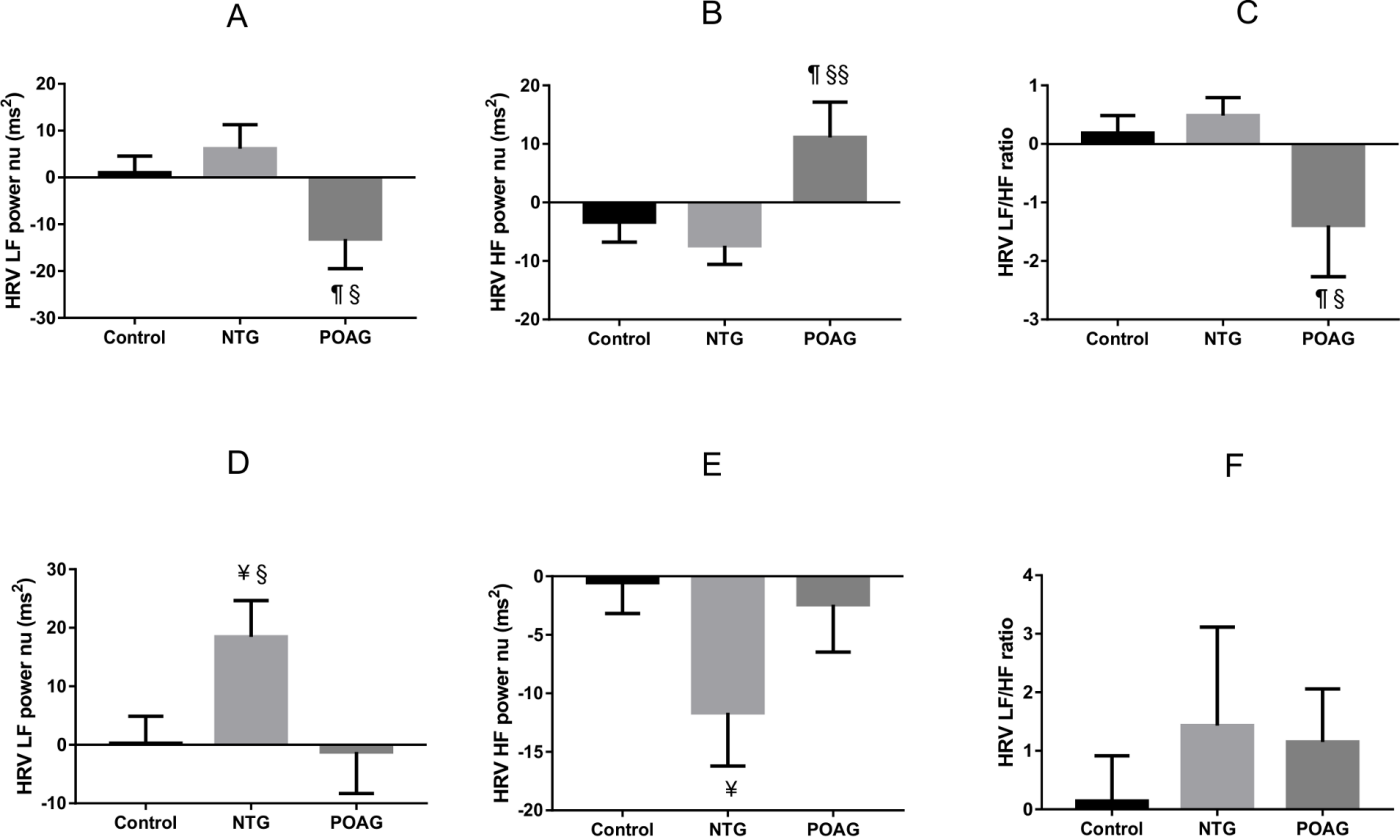
Group comparison of HRV parameter changes to meal ingestion. **A, B, C** shows, in supine position, from pre-ingestion to early after meal ingestion (30min postprandially), HRV parameters’ changes are different in POAG, compared to NTG and Control. There are reductions in HRV LF power nu and HRV LF/HF ratio, and an increase in HRV HF power nu in POAG. **D, E, F** shows, in standing position, from fasting to 90 minutes after carbohydrate ingestion (maximal response), HRV parameters’ changes are different in NTG, compared to POAG and Control. There is an increase in HRV LF power nu and a decrease in HRV HF power nu in NTG. § *P*< 0.05, §§ *P*< 0.01, NTG vs POAG; *P*< 0.05, Control vs POAG; ¥ *P*< 0.05, Control vs NTG; data shown as Mean ± SEM.

Early after eating (30min), in the supine position, changes in HRV LF power nu, HRV HF power nu, and HRV LF/HF ratio were different for group comparisons: POAG vs control (all *P*<0.05), and POAG vs NTG (*P*<0.05, 0.01, 0.05) (Fig 7); whereas these changes in HRV parameters were similar between control and NTG subjects (*P* = 0.41, 0.45, 0.53) (Fig 7). In the standing position, no difference was found between group comparisons for early postprandial changes in the above HRV parameters.

Later after carbohydrate ingestion (maximal response at 90min), in the standing position, changes in HRV LF power nu were different for NTG vs control (*P*<0.05), and NTG vs POAG (*P*<0.05); changes in HRV HF power nu was different between NTG and control (*P*<0.05), but similar for control vs POAG and NTG vs POAG (*P* = 0.69, 0.13); changes in HRV LF/HF ratio was not different between group comparison (Fig 7). In supine position, no difference was found between groups in the above HRV parameters’ changes.

(See S1 Table for HRV, BPV and BRS parameters in absolute and normalised units in controls, NTG and POAG, from fasting state to over time postprandial state in both supine and standing positions.)

## Discussion

The new findings of this study are that while glaucoma patients were found to manifest features of systemic autonomic dysregulation, there was a difference in the characteristics of the response to carbohydrate ingestion in different sub-types of glaucoma, and this was irrespective of insulin resistance. RNFL thickness is positively correlated to autonomic response to carbohydrate ingestion; attenuated autonomic responses may be associated with RNFL loss, i.e. advanced glaucomatous disease [42]. In the early postprandial state, POAG patients showed autonomic failure with associated hypotension (mean arterial pressure). Later following carbohydrate ingestion, NTG patients exhibited cardiac vagal withdrawal and sympathetic over-activation (Table 5. A summary for study findings). The current study provides new experimental evidence that *differential* global autonomic dysregulation may underlie, and correlate with, the pathogenesis of vascular mediated changes in NTG and POAG.

**Table 5:** Abnormal autonomic responses to laboratory challenges and their clinical relevance in POAG and NTG.

Laboratory challenges	Autonomic responses in POAG	Autonomic responses in NTG	Clinical relevance
**1**, hemodynamic perturbation induced by splanchnic hyperaemia and peripheral vasodilation early after meal ingestion	Autonomic failure, i.e., vagal enhancement and sympathetic inhibition, with associated postprandial hypotension	comparable to Controls	Food intake is an essential normal daily activity. This autonomic failure associated depressor response may result in unstable ocular perfusion in the postprandial state in POAG.
**2**, hyperinsulinemia mediated sympathetic overexcitation after carbohydrate ingestion—central action of insulin	comparable to Controls	Vagal withdrawal and sympathetic hyper-responsiveness	Carbohydrate ingestion may trigger excessive vagal inhibition and sympathetic excitation in NTG, contributing to endothelial dysfunction, e.g., higher Endothelin-1 level.
**3**, orthostatic stress induced hemodynamic changes	Attenuated autonomic responses compared to Controls	Attenuated autonomic responses compared to Controls	This impaired baroreflex-mediated autonomic dysfunction in both POAG and NTG may chronically disturb cerebral / ocular blood flow.

### Postprandial autonomic failure with depressor response in POAG

Shortly after eating (30min time point), there is a significant depressor response (a reduction in MAP of approx. 10mmHg), accompanied with cardiac paradoxical sympathovagal response in POAG patients, i.e. sympathetic inhibition and vagal enhancement [43]. In contrast, in both NTG and control subjects, mean arterial pressure was stable with a functional cardiac autonomic response. Indeed, the postprandial hypotensive effect in POAG patients may directly cause unstable ocular perfusion as seen in patients with autonomic failure [44]. A previous study reported that cold provocation that induced a normal pressor response in control subjects was accompanied by stable ocular blood flow, whereas in POAG patients, the blood pressure failed to increase accompanied with a significant decrease in ocular blood flow, suggesting a concurrent systemic autonomic failure and instability of ocular blood flow in POAG [9]. The current study supports this idea with experimental demonstration of autonomic dysfunction and provides new evidence that POAG patients may undergo repetitive systemic autonomic failure in the postprandial state during daily life; a situation that is perhaps more physiologically relevant than the cold pressor test.

After meal ingestion, splanchnic hyperaemia and vascular dilatation peak in the early, but not the later phase, of the postprandial state [22, 45]. The autonomic nervous system regulates postprandial meal ingestion-mediated hemodynamic perturbation; a failure of autonomic regulation may lead to postprandial hypotension: most commonly in the elderly and in patients with dysautonomia [46]. Similar to orthostasis-induced venous pooling, postprandial splanchnic vasodilation and hyperaemia may induce baroreceptor and cardiopulmonary mechanoreceptor (volume receptor) overstimulation, and as a consequence an excessive sympathetic outflow in patients with syncope experience [47]. Indeed, following head-up tilt, cardiac sympathetic inhibition and vagal predominance were observed as a sign preceding vasovagal syncope [43]. We here observed a coupling of efferent cardiac sympathetic inhibition and vagal enhancement soon after eating in POAG subjects, suggesting a paradoxical sympatho-vagal response to hemodynamic perturbation. This autonomic failure may be subclinical since none of the subjects / patients exhibited clinical symptoms and signs of hypo-perfusion during the experiments. We postulate that, in POAG patients, cardiac autonomic failure may contribute to a depressor response [47], leading to an unstable ocular perfusion in the postprandial state.

### Postprandial sympathetic hyper-responsiveness and vagal inhibition in NTG

Another major finding is that following carbohydrate ingestion, compared to age- and gender- matched control subjects, there is a cardiac sympathetic activation and vagal inhibition in NTG patients in the later phase of the postprandial state.

As is well-known, carbohydrate ingestion induces increases in plasma insulin level with associated sympathetic responses [24, 48, 49]. Food intake is controlled by the central nervous system [27]. Within the hypothalamus, the arcuate nucleus is the key site [25] that mediates central sympathoexcitatory actions of insulin [50]. Sympathetic over-activation following carbohydrate ingestion (hyperinsulinemia) is seen in patients with cardiovascular disorders, such as hypertension [51], metabolic syndrome and pre-diabetes [10–12]. Furthermore, carbohydrate ingestion induced vagal inhibition is also recognised as a cardiovascular risk factor [18]. The current study, for the first time, demonstrates an abnormal sympathetic hyper-responsiveness and vagal inhibition following carbohydrate ingestion in NTG, irrespective of insulin resistance. Recent evidence shows that calorie restriction may counteract the incidence of glaucoma [16, 17]. In western society, humans spend most of their awake hours in a fed state, our finding may explain the excessive sympathetic nerve activity observed during 24 hours ECG recordings in NTG patients [20]. Autonomic dysfunction has been associated with faster central visual field defect progression in NTG patients [21].

Since meal ingestion is an essential normal daily behaviour, abnormal sympathetic activation may trigger endothelial dysfunction [52]. Evidence of endothelial dysfunction was observed in NTG patients, including higher basal plasma endothelin-1 and abnormal endothelin-1 response to laboratory stimuli [53, 54]. In addition, chronic ocular administration of endothelin-1 can induce ocular ischemia glaucomatous optic neuropathy in primates [55].

### Autonomic dysfunction during postural stress in both NTG and POAG

Our study also demonstrates that NTG and POAG exhibited impaired autonomic responses to postural stress in both the fasting and postprandial states: a finding that is consistent with previous studies that report that both POAG and NTG patients manifest an impaired baroreflex-mediated autonomic dysfunction to neck suction [19].

A blunted cardiac sympathovagal response to orthostatic stress occurs in aging populations [56], essential hypertensive patients [57], pre-hypertensive humans [14] and in chronic psychosocial stress [13]. Autonomic dysfunction can be a harbinger of cardiovascular ischemic events [11–14, 58]. Assuming an upright posture is a normal daily activity and induces a redistribution of the cardiac output. Although cerebral autoregulation compensates for changes in posture, perfusion in the brain may still be challenged in susceptible individuals [59]. Impaired cardiac output in human (MAP<80mmHg) may also jeopardise ocular perfusion, as seen in patients with autonomic failure exhibiting a significant decrease in ocular perfusion while standing up [44]. It is therefore conceivable that cardiac autonomic dysfunction may chronically disturb cerebral blood flow and cause unstable ocular blood flow. It has previously been reported that vascular dysregulation, i.e. the instability of ocular blood flow underlies the pathogenesis of both NTG and POAG [3, 60].

### Limitations

Topical beta-blockers are known to depress nocturnal arterial blood pressure which potentially could exacerbate glaucoma [61]. Some glaucoma subjects were taking beta-blocker eye drops in our study; we asked subjects to discontinue these drops overnight prior to the study. While there may be still some residual systemic beta-blockade in these subjects, we believe that topical beta-blockers did not affect our overall study results. Firstly the control, NTG and POAG subjects all showed comparable baseline hemodynamic and autonomic parameters, and similar magnitude of heart rate increases in response to meal ingestion and postural stress. Secondly nocturnal BP measurement is different to our laboratory stimuli in regard to evaluating dynamic autonomic responses. In our experimental design [29], splanchnic hyperaemia induced early-postprandial hemodynamic perturbation [22] evokes significant baroreflex-mediated autonomic responses, similar to postural challenge. A meal ingestion effect would therefore be unlikely to be masked by a topical beta-blocker. In addition, we observed postprandial autonomic failure with associated hypotension in POAG, with HR increased, but not decreased [46]. This is inconsistent with those studies concerning endogenous circadian rhythm related nocturnal hypotension in glaucoma, with concurrent reduction in BP and HR due to drug adverse effect [61]. (3) In the later postprandial state, we find that carbohydrate ingestion enhances cardiac sympathetic response in NTG, which is clearly not consistent with a drop related sympathetic inhibitor effect.

The current experimental protocol may provide opportunities for future experimental study and risk profiling of glaucoma patients. There is an opportunity to conduct a longitudinal follow-up study for the association of systemic vascular risk and progression of glaucomatous optic neuropathy, in newly-diagnosed glaucoma patients. The correlation of glaucoma severity and autonomic responses to laboratory stimuli could also be studied in more detail.

## Conclusion

The current study demonstrates that POAG patients manifested a cardiac autonomic failure associated with a significant depressor response soon after eating. Following carbohydrate ingestion, NTG patients exhibited a cardiac sympathetic hyper-responsiveness and vagal inhibition in the later phase of the postprandial state, irrespective of insulin resistance. In addition, both NTG and POAG showed blunted cardiac autonomic responses to postural challenge. Therefore, the current study provides important experimental evidence that glaucoma is associated with a systemic cardiovascular disorder. It provides a new mechanistic insight that the distinct autonomic dysregulation may underlie, or correlate with, the pathogenesis of the two forms of glaucoma. Clinically, it is worth noting that the assessment procedure is simple and non-invasive, and may be useful as a tool for monitoring responses to treatment in individual patients.

## Supporting information

S1 Table Overall hemodynamic and autonomic changes to meal ingestion and postural stress in control, NTG and POAG subjects. (PDF)

## Abbreviations

MAP: mean arterial pressure
HR: heart rate
RR interval: the interval between successive peaks of R wave in ECG
LF: low frequency
HF: high frequency
Nu: normalised units
HRV: heart rate variability
HRV LF power: RR interval low frequency spectral power
HRV LF power nu: RR interval low frequency spectral power in normalised units
HRV HF power: RR interval high frequency spectral power
HRV HF nu: RR interval HF spectral power in normalised units
HRV LF/HF ratio: the ratio of HRV LF power / HRV HF power
SBP LF power: systolic blood pressure variability low frequency spectral power
BRS: sequence method for spontaneous baroreflex sensitivity retinal nerve fibre layer (RNFL)
