# Supplementary material for: Carbohydrate ingestion induces differential autonomic dysregulation in normal-tension glaucoma and primary open angle glaucoma
Source: PLoS One. 2018 Jun 7;13(6):e0198432. doi: 10.1371/journal.pone.0198432 (PMC5991678; doi:10.1371/journal.pone.0198432)
Supplement: S1 Table — (PDF) [file pone.0198432.s001.pdf]

Control		0 minute	30 minutes	60 minutes	90 minutes	120 minutes	*P* value	
							posture	ingestion
MAP (mmHg)	supine	90.2±2.4	86.3±2.2	88.9±2.0	88.6±2.0	87.5±2.0	0.0005**	0.24
	standing	92.4±2.8	92.5±2.7	96.4±2.4	94.4±2.3	97.2±2.9		
HR (bpm)	supine	60.6±1.1	65.4±1.3	66.8±1.4	66.5±1.2	65.7±1.2	<0.0001**	<0.0001**
	standing	69.6±1.4	72.7±1.5	75.2±1.6	76.8±1.7	76.2±1.4		
HRV LF power (ms^2)	supine	647.0±157.0	883.5±306.3	641.1±138.7	577.7±126.2	411.0±70.2	0.13	0.72
	standing	512.0±108.7	390.3±75.2	349.4±66.2	345.0±63.7	673.5±314.8		
HRV LF nu	supine	52.8±3.7	53.8±3.59	53.8±3.8	52.1±3.8	54.4±3.5	0.002**	0.95
	standing	61.5±4.4	61.3±3.6	63.3±3.8	61.8±3.6	63.4±3.4		
HRV HF power (ms^2)	supine	603.9±204.4	823.2±241.9	622.3±178.2	523.4±151.0	400.1±106.5	0.36	0.29
	standing	668.4±326.4	473.3±272.6	311.2±116.3	221.7±62.8	595.4±416.0		
HRV HF nu	supine	41.3±3.0	37.9±2.7	35.7±2.5	37.3±2.5	36.9±2.6	0.0001**	0.47
	standing	29.5±3.0	30.0±2.6	28.4±2.6	29.0±2.7	27.2±2.2		
HRV LF/HF ratio	supine	1.8±0.2	2.0±0.3	2.1±0.3	2.0±0.3	2.1±0.3	0.0012**	0.71
	standing	4.0±0.7	3.5±0.6	4.7±1.1	4.2±1.0	3.9±0.7		
BRS (ms/mmHg)	supine	14.2±1.8	14.9±2.9	9.9±0.8	11.2±1.6	9.2±0.8	<0.0001**	0.0034**
	standing	9.0±1.9	8.2±1.2	5.9±0.7	6.7±0.8	5.6±0.5		
SBP LF power ($mmHg^2$)	supine	7.2±1.1	9.5±1.8	11.5±1.5	13.6±2.2	8.9±1.3	<0.0001**	0.015*
	standing	21.3±2.9	23.7±3.6	23.8±3.8	29.2±4.6	30.1±4.7		
**NTG**		**0 minute**	**30 minutes**	**60 minutes**	**90 minutes**	**120 minutes**	***P* value**	
							**posture**	**ingestion**
MAP (mmHg)	supine	91.0±2.6	88.5±2.7	90.7±2.9	91.7±2.3	88.0±2.6	0.08	0.42
	standing	91.8±2.7	92.3±4.0	96.5±2.6	94.5±1.9	88.9±4.5		
HR (bpm)	supine	56.2±1.5	61.3±2.0	62.5±2.1	63.7±1.9	63.6±1.8	<0.0001**	<0.0001**
	standing	63.9±2.2	68.7±2.5	70.8±2.6	73.6±2.7	71.4±2.5		
HRV LF power (ms^2)	supine	499.6±167.7	380.7±95.7	380.6±93.3	481.4±237.1	491.4±150.6	0.59	0.48
	standing	448.3±78.0	940.7±392.4	422.6±102.5	425.9±97.3	416.4±147.3		
HRV LF nu	supine	47.8±4.9	53.9±4.7	57.0±5.3	50.2±5.3	53.8±6.1	0.03*	0.04*
	standing	49.8±5.8	50.4±6.1	57.9±4.8	69.8±4.2	63.8±5.4		
HRV HF power (ms^2)	supine	509.7±129.3	323.5±81.4	415.2±162.5	686.4±334.7	568.2±221.8	0.53	0.85
	standing	534.5±204.7	581.9±205.0	255.9±91.1	145.6±52.0	464.3±324.8		
HRV HF nu	supine	45.2±3.8	37.8±4.0	35.5±3.8	40.2±3.6	37.2±4.6	0.003**	0.006**
	standing	38.1±4.1	34.2±3.7	29.8±3.1	24.9±3.9	27.9±4.2		
HRV LF/HF ratio	supine	1.4±0.3	1.9±0.3	2.5±0.6	1.8±0.4	2.4±0.5	0.03*	0.03*
	standing	2.3±0.7	2.5±0.6	4.0±1.4	5.0±1.1	4.7±1.2		
BRS (ms/mmHg)	supine	16.4±3.3	9.4±1.4	10.4±2.0	6.3±0.5	9.3±1.6	0.008**	0.01*
	standing	7.3±1.0	8.4±1.1	6.8±1.3	5.0±0.6	8.7±3.7		
SBP LF power ($mmHg^2$)	supine	11.1±2.4	6.7±1.3	11.6±1.3	12.1±1.6	16.5±3.4	0.002**	0.09
	standing	19.6±2.7	19.4±3.5	22.7±3.4	25.2±4.4	24.5±3.7		

POAG		0 minute	30 minutes	60 minutes	90 minutes	120 minutes	*P* value	
							**posture**	**ingestion**
MAP (mmHg)	supine	97.3±3.1	86.7±5.4	95.9±4.3	96.0±3.7	92.9±2.7	0.03*	0.01*
	standing	100.5±3.6	93.4±4.8	100.6±4.2	100.5±3.2	99.8±2.7		
HR (bpm)	supine	58.1±1.5	64.0±1.8	64.8±2.0	65.1±1.6	65.0±1.4	<0.0001**	<0.0001**
	standing	69.1±2.1	69.8±2.4	76.2±2.6	79.0±2.6	77.0±2.3		
HRV LF power (ms^2)	supine	1077.3±415.3	536.5±253.3	1041.4±425.6	599.1±174.9	431.1±85.7	0.15	0.84
	standing	258.3±62.2	806.5±430.2	335.2±78.9	371.6±93.9	637.2±167.1		
HRV LF nu	supine	59.3±5.3	46.1±5.6	56.9±4.4	59.1±5.0	56.0±5.9	0.14	0.13
	standing	62.9±4.8	55.5±5.4	61.7±6.6	61.6±5.8	65.8±6.1		
HRV HF power (ms^2)	supine	777.8±397.3	939.7±455.5	788.7±297.8	415.6±141.3	469.4±214.7	0.30	0.26
	standing	131.5±37.1	961.2±531.2	278.9±116.5	301.5±138.2	772.1±327.8		
HRV HF nu	supine	35.5±4.3	46.6±4.1	37.4±3.3	35.7±4.2	34.4±3.8	0.0002**	0.005**
	standing	26.4±3.0	33.6±4.2	24.9±3.2	24.0±3.0	22.9±3.0		
HRV LF/HF ratio	supine	2.3±0.5	1.6±0.4	2.3±0.6	2.8±0.6	2.3±0.5	0.01*	0.06
	standing	3.5±0.5	2.7±0.7	4.3±1.2	4.6±1.1	5.4±1.4		
BRS (ms/mmHg)	supine	11.9±1.6	15.4±2.9	10.9±1.7	10.2±1.8	8.6±1.0	0.002**	0.002**
	standing	7.7±1.9	8.6±2.2	5.1±0.7	4.2±0.6	4.6±0.6		
SBP LF power ($mmHg^2$)	supine	9.6±2.1	7.5±1.5	14.9±4.4	13.9±4.8	14.8±3.6	0.003**	0.0004**
	standing	16.2±2.3	18.9±6.6	36.6±8.1	29.0±5.4	29.0±2.9		

Two-way ANOVA of repeated measures, data shown as Mean±SEM. Statistical significance defined as $P<0.05$. * $P<0.05$; ** $P<0.01$.
